# Adapting Mobile Beacon-Assisted Localization in Wireless Sensor Networks

**DOI:** 10.3390/s90402760

**Published:** 2009-04-20

**Authors:** Guodong Teng, Kougen Zheng, Wei Dong

**Affiliations:** College of Computer Science, Zhejiang University, Hangzhou 310027, P.R. China; E-Mails: zkg@zju.edu.cn; dongw@zju.edu.cn

**Keywords:** Wireless Sensor Networks (WSNs), Localization, Mobile Beacon-assisted Localization (MBL), Adapting Mobile Beacon-assisted Localization (A-MBL), Particle filter

## Abstract

The ability to automatically locate sensor nodes is essential in many Wireless Sensor Network (WSN) applications. To reduce the number of beacons, many mobile-assisted approaches have been proposed. Current mobile-assisted approaches for localization require special hardware or belong to centralized localization algorithms involving some deterministic approaches due to the fact that they explicitly consider the impreciseness of location estimates. In this paper, we first propose a range-free, distributed and probabilistic Mobile Beacon-assisted Localization (MBL) approach for static WSNs. Then, we propose another approach based on MBL, called Adapting MBL (A-MBL), to increase the efficiency and accuracy of MBL by adapting the size of sample sets and the parameter of the dynamic model during the estimation process. Evaluation results show that the accuracy of MBL and A-MBL outperform both Mobile and Static sensor network Localization (MSL) and Arrival and Departure Overlap (ADO) when both of them use only a single mobile beacon for localization in static WSNs.

## 1. Introduction

Wireless Sensor Networks (WSNs) are composed of large numbers of tiny sensor devices with wireless communication capabilities. WSN systems have been developed recently for numerous applications such as military surveillance [1], environmental monitoring [2,3], target tracking [4,5], habitat monitoring [6] and structural monitoring [7], etc. Because many of them require sensor position information, *localization* has been an important problem in WSNs [8] and several localization systems and algorithms have been proposed in the past. The large body of solutions for the sensor node localization problem can be categorized based on whether the localization techniques are *Range-based* or *Range-free*, whether the localization algorithms are *Centralized* or *Distributed*, and whether localization results are *Deterministic* or *Probabilistic*.

In most existing WSNs, sensors are static [9]. The localization of these *static WSNs* relies on several beacons which know their locations scattered throughout the sensor networks and the precision of the localization increases with the number of beacons. The main problem with an increased number of beacons is that they are more expensive than the rest of the sensor nodes, and after these sensor nodes have been localized, the beacons become useless. The leads us to believe that a single mobile beacon which can travel the entire deployment region based on some traverse route can be used to help localize the entire network. Using a single mobile beacon that knows its position is broadly equivalent to using many static beacons each broadcasting once.

In this paper, we propose two mobile beacon-assisted localization approaches, *Mobile Beacon-assisted Localization* (MBL) and Adapting MBL (A-MBL) for static WSNs. Compared to some proposed mobile-assisted approaches [10–12], MBL and A-MBL do not require any specially designed hardware due to the range-free technique employed. Compared to the algorithm requiring the gathering of connectivity data (range or proximity) from the network to a more computationally powerful device [13], MBL and A-MBL focus on distributed algorithms. As the approaches described in [14,12], MBL and A-MBL adopt probabilistic methods which give an area where a sensor might reside, along with the likelihood of such an estimate. Like the Arrival and Departure Overlap (ADO) approach [15], MBL and A-MBL rely on direct Arriver and Leaver information from a single mobile-assisted beacon. Especially, inspired by ideas from [16,17], we adopt an adapting mechanism to improve the efficiency and accuracy of MBL.

This paper offers the following two major contributions:

- We propose a range-free, distributed and probabilistic MBL approach. This approach outperforms both Mobile and Static sensor network Localization (MSL) and ADO when both of them use only a single mobile beacon for localization in static WSNs.
- We propose another approach based on MBL, called A-MBL, to increase the efficiency and accuracy of MBL by adapting the size of sample sets and the parameter of the dynamic model during the estimation process.

The rest of this paper is organized as follows: Section 2 defines mobile beacon-assisted localization problem from Bayesian filter and particle filter perspective. Section 3 presents details of the proposed MBL and A-MBL algorithms. Section 4 shows and discusses our evaluation results. Section 5 gives an overview of related works. Finally, Section 6 concludes our work.

## 2. Description of the Problem

### 2.1. Mobile Beacon-assisted Localization Problem

Let us consider a sensor network with *M* static sensor nodes in a 2D plane which do not have *a priori* known locations (called *unknown nodes*) and a single mobile node (called *beacon*), equipped with localization hardware, e.g., GPS, which allows it to know its location at all times. After random deployment of the unknown nodes in a fixed-size area, the beacon traverses the sensor network while broadcasting packets which contain the coordinates of itself and other information. Any unknown node receiving these packets from the beacon (called *observation*) can recognize that it is in the area around the beacon’s current location with a certain probability. With each observation in a series of different time, the unknown node’s location is bounded in the beacon’s transmission area. The accuracy can be improved when the unknown node obtains more observations from the beacon. Location estimates and observations are assumed to be available at discrete times. For dynamic state estimation, the discrete-time approach is widespread and convenient [18].

### 2.2. Problem Description with Bayesian Filter

If we solve the above-mentioned localization problem with a probabilistic approach, we are interested in estimating the unknown node’s *real location R* at the current time-step *t*, given knowledge about the initial location estimate and all observations *O*_1:*t*_ = {*O*_1_,…,*O_t_*} up to the current time *t*. This localization problem is an instance of the *Bayesian filtering* problem which requires the estimation of the state of a system that changes over time using a sequence of noisy measurements made on the system [18], where we are interested in constructing the posterior density *p*(*l_t_*| *O*_1:*t*_) of the current location estimate *l_t_* conditioned on all observations *o*_1: *t*_ from the beacon. To define this localization problem from a Bayesian filter perspective, we assume that we have an *initial distribution*, a *dynamic model* and an *observation model*:

(1) $$p(l_{0}|o_{0}),$$

(2) $$l_{t}=f(l_{t-1})\;\;\text{for}\;\;\;\;\;\;\;t\geq 1,$$

(3) $$o_{t}=g(l_{t})\;\;\;\;\;\;\;\;\text{for}\;\;\;\;\;\;\;t\geq 1.$$

By Equation (2) and Equation (3) we mean that *l_t_* and *o_t_* are assumed to be generated by functions *f*(*·*) and *g*(*·*), respectively. The precise form of the functions implies via a change of variables the transition probability density *p*(*l_t_*|*l*_0:*t*_, *o*_1:*t*−1_) and the observation probability density *p*(*o_t_*|*l*_0:*t*_, *o*_1:*t*−1_). We denote by *l*_0:*t*_ = {*l*_0_,…,*l_t_*}, the location estimate up to time *t*. Note that we could assume Markov transitions and conditional independence to simplify the model due to the constraints in computing and memory power of the sensor node, i.e. current location estimate *l_t_* is only dependent on the previous location estimate *l*_*t*−1_ and the current observation *o_t_* is only dependent on the current location estimate *l_t_*, then the dynamic model *p*(*l_t_*|*l*_0:*t*−1_*, o*_1:*t*−1_) = *p*(*l_t_*|*l*_*t*−1_) and the observation model *p*(*o_t_*|*l*_0:*t*_, *o*_1:*t*−1_) = *p*(*o_t_*|*l_t_*). The posterior density *p*(*l_t_*|*o*_1:*t*_) = *p*(*l_t_*|*o_t_*) may be obtained after *initialization*, recursively, in two stages: a *prediction* stage and an *update* stage.

**Initialization**: It is assumed that the initial *p*(*l*_0_|*o*_0_) ≡ *p*(*l_o_*) of the location estimate, which is also known as the prior density, is available (*o*_0_ being the set of no observations).

**Prediction**: Suppose that the posterior density *p*(*l_t_*_−1_|*o*_*t*−1_) at time *t*−1 is available. The prediction stage involves using a dynamic model Equation (2) to obtain the prior density *p*(*l_t_*|*o*_*t*−1_) of the location estimate at time *t* via the Chapman-Kolmogorov equation:

(4) $$p(l_{t}|o_{t-1})=\int p(l_{t}|l_{t-1})p(l_{t-1}|o_{t-1})\;{\mathrm{dl}}_{t-1}.$$

**Update**: At time step *t*, an observation *o_t_* becomes available, and this may be used to update the prior density via Bayes’ rule:

(5) $$p(l_{t}|o_{t})=\frac{p(o_{t}|l_{t})p(l_{t}|o_{t-1})}{p(o_{t}|o_{t-1})}$$

where the normalizing constant

(6) $$p(o_{t}|o_{t-1})=\int p(o_{t}|l_{t})p(l_{t}|o_{t-1})\;{\mathrm{dl}}_{t}$$

depends on the likelihood function *p*(*o_t_*|*l_t_*) defined by the observation model Equation (3). In the update stage Equation (5), the observation *o_t_* is used to modify the prior density to obtain the required posterior density of the current location estimate.

### 2.3. Problem Description with Particle Filter

To address the complexity of the integration step in Bayesian filter, many optimal or suboptimal approaches are proposed. The recurrence relations Equation (4) and Equation (5) form the basic for the optimal or suboptimal Bayesian solution. Solutions do exist in a restrictive set of cases [18], including the *Kalman filter* (optimal Bayesian filter) and *particle filter* (suboptimal Bayesian filter) which approximates the optimal Bayesian solution, etc.

The Kalman filter assumes that the posterior density at every time step is Gaussian and, hence, parameterized by a mean and covariance, provided that certain assumptions hold: *f*(*·*) and g(·) in the dynamic model and the observation model are known and also are a linear function. In many situation of interest, such as mobile beacon-assisted localization problem in our scenarios, the assumptions made above do not hold. The Kalman filter cannot, therefore, be used as described – approximations are necessary.

The particle filter solutions offer a number of significant advantages compared with other techniques currently available, including the Kalman filter. These advantages arise principally from the generality of the approach, which allows inference of full posterior distributions in general state-space models, which may be both nonlinear and non-Gaussian.

Thus, in this paper, we use a particle filter (also called *sequential Monte Carlo method*) to perform a Bayesian filter on a sample representation. The key idea is to represent the required posterior density by a set of random samples with associated weights and to compute estimates based on these samples and weights [18]. As the number of samples becomes very large, this characterization of particle filter becomes an equivalent representation to the usual functional description of the posterior density, and the particle filter approaches the suboptimal Bayesian estimate.

In order to develop the details of the algorithm, let $\left\{<l_{t}^{i},w_{t}^{i}>,i=1,...,N\right\}$ denotes a *random measure* that characterizes the posterior density *p*(*l_t_*|*o_t_*), where $L_{t}=\{l_{t}^{i},i=1,...,N\}$, is a set of support *samples* (or called *particles*) with associated *weights* $W_{t}=\{w_{t}^{i},i=1,...,N\}$. The weights are normalized such that $\sum_{i=1}^{N}\;w_{t}^{i}=1$. Then, the posterior density at *t* can be approximated as:

(7) $$p(l_{t}|o_{t})\approx \sum_{i=1}^{N}\;w_{t}^{i}\delta (l_{t}-l_{t}^{i})$$

where *δ*(·) is Dirac delta function

(8) $$\delta (l_{t}-l_{t}^{i})=\{\begin{array}{c} 1\;\;\;\;\;\;\mathrm{if}\;\;\;l_{t}=l_{t}^{i}\\ 0\;\;\;\;\;\;\;\mathrm{otherwise} \end{array}$$

samples *l^i^* are easily generated from a *proposal density* (or called *importance density*) *q*(*·*):

(9) $$l^{i}∼q(l)$$

where the symbol ∼ denotes sample generated sign, i.e., the samples on the left side are generated from the probability density on the right side. Weights are defined by:

(10) $$w_{t}^{i}\propto \frac{p(l_{t}^{i}|o_{t})}{q(l_{t}^{i}|o_{t})}$$

where the symbol ∝ is used to denote proportionality up to a normalization constant.

The proposal density is chosen to factorize such that:

(11) $$q(l_{t}|o_{t})=q(l_{t}|l_{t-1},o_{t})q(l_{t-1}|o_{t-1})$$

To derive the weight update equation, *p*(*l_t_*|*o_t_*) from Equation (5) is expressed via Bayes’ rule:

(12) $$\begin{array}{ll} p(l_{t}|o_{t}) & =\frac{p(o_{t}|l_{t})p(l_{t}|o_{t-1})}{p(o_{t}|o_{t-1})}\\ & \propto \frac{p(o_{t}|l_{t})p(l_{t}|l_{t-1})p(l_{t-1}|o_{t-1})}{p(o_{t}|o_{t-1})}\\ & \propto p(o_{t}|l_{t})p(l_{t}|l_{t-1})p(l_{t-1}|o_{t-1}). \end{array}$$

The weight update equation can then be shown to be (the proposal density is chosen to be dynamic model Equation (2), i.e. $q(l_{t}^{i}|l_{t-1}^{i},o_{t})=p(l_{t}^{i}|l_{t-1}^{i})$):

(13) $$\begin{array}{ll} w_{t}^{i} & \propto \frac{p(l_{t}^{i}|o_{t})}{q(l_{t}^{i}|o_{t})}\\ & \propto \frac{p(o_{t}|l_{t}^{i})p(l_{t}|l_{t-1}^{i})p(l_{t-1}^{i}|o_{t-1})}{q(l_{t}^{i}|l_{t-1}^{i},o_{t})q(l_{t-1}^{i}|o_{t-1})}\\ & \propto \frac{p(o_{t}|l_{t}^{i})p(l_{t}^{i}|l_{t-1}^{i})}{q(l_{t}^{i}|l_{t-1}^{i},o_{t})}w_{t-1}^{i}\\ & \propto p(o_{t}|\;\;\;l_{t}^{i})w_{t-1}^{i}. \end{array}$$

The generic particle filter proceeds for localization are as follows:

**Initialization**: *N* samples and weights are chosen from the initial distribution Equation (14) and the initial observation Equation (15), respectively.
  (14) $$l_{0}^{i}∼p(l_{0}|o_{0}),$$
  (15) $$w_{0}^{i}=p(o_{0}|l_{0}^{i}).$$
**Prediction**: It starts from the set of samples $\left\{l_{t-1}^{i},i=1,...,N\right\}$ computed in the previous iteration, and applies the dynamic model to each sample $l_{t-1}^{i}$ by sampling from the density *p*(*l_t_*|*l_t_*_−1_), i.e. for each particle $l_{t-1}^{i}$ draw one sample $l_{t}^{i}$ from *p*(*l_t_*|*l_t_*_−1_) by:
  (16) $$l_{t}^{i}∼p(l_{t}|l_{t-1}).$$
**Update**: It takes into account the observation *o_t_*. Each weight $w_{t}^{i}$ of the sample in $\left\{l_{t}^{i},i=1,...,N\right\}$ is obtained by the importance weight Equation (13), i.e. the likelihood of $\left\{l_{t}^{i},i=1,...,N\right\}$ given *o_t_*.

## 3. Mobile Beacon-Assisted Localization

### 3.1. MBL

**Assumption**: We assume that all unknown nodes are randomly deployed in an area of size *S* and each sensor (unknown node or beacon) has the same ideal radio range *r*. The algorithm does not assume very tightly synchronized clocks. The beacon is capable of moving a distance *v_b_* in a time step in any direction where 0 ≤ *v_b_* ≤ *v_max_*. The beacon knows *v_max_*, but it does not know the value of *v_b_* or the direction of movement in any time step. At time *t*, every unknown node within the radio range of the beacon will hear a location announcement from that beacon. In a realistic deployment, it would be necessary to deal with network collisions and account for missed messages [19].

**Initialization**: In this stage, all unknown nodes have no information about their locations. The initial set of samples Equation (17) for each unknown node is chosen randomly from the whole deployed area and represented by a set of uniformly distributed samples with equal weights Equation (18). The weight equal to one represents the importance of corresponding sample, which infers one of the location estimates of the unknown node. The beacon’s current initial position Equation (19) is also chosen randomly from the whole deployment area. The beacon’s previous initial position (for further use) is chosen randomly from out of the deployment area and out of the radio range of any unknown node Equation (20):

(17) $$L_{0}=\{l_{0}^{i}|l_{0}^{i}∼p(l_{0})=\frac{1}{S},\;\;1\leq i\leq N\},$$

(18) $$w_{0}^{i}=1,\;\;\;\;1\leq i\leq N,$$

(19) $${\mathrm{beacon}}_{c}∼\frac{1}{S},$$

(20) $${\mathrm{beacon}}_{p}=({\mathrm{beacon}}_{p}∼\frac{1}{\bar{S}})\wedge (d({\mathrm{beacon}}_{p},R)>r).$$

where *L_0_* denotes the initial set of each sample, $w_{0}^{i}$ denotes the initial weight of each sample, *beacon_c_* denotes the beacon’s current location, *d(beacon_p_,R)* denotes the distance between locations *beacon_p_* and *R*, *R* denotes the unknown node’s real location, *S̄* denotes the area out of the deployment area, and *beacon_p_* represents the beacon’s previous location.

**Prediction**: In this stage, we adopt a dynamic model that the unknown node is capable of moving a distance *v_node_* in a time step in any direction where 0 ≤ *v_node_* ≤ *α*. The unknown node knows *v_node_*, but it does not know the value of *v_node_* or the direction of movement in any time step. Then, the unknown node generates new samples as follows:

(21) $$P_{t}=\{l_{t}^{i}|l_{t}^{i}\;\text{is selected from}\;p(l_{t}^{i}|l_{t-1}^{i}),\;\text{where}\;l_{t-1}^{i}\in L_{t-1}\;\text{for all}\;1\leq i\leq N\},$$

where *P_t_* represents approximation of prior density at time *t* after prediction stage, *L*_*t*−*1*_ represents approximation of posterior density at time *t*−*1*, and the transition equation for each sample described as follows:

(22) $$p(l_{t}|l_{t-1})=\{\begin{array}{c} \begin{array}{c} \frac{1}{\pi \alpha^{2}}\;\;\;\;\;\;\;\;\mathrm{if}\;\;\;\;\mathrm{filter}(R)=\mathrm{TRUE}\\ \;\;\;0\;\;\;\;\;\;\;\;\;\;\;\;\;\;\;\;\;\;\;\;\mathrm{if}\;\;\mathrm{filter}(R)=\mathrm{FALSE}. \end{array} \end{array}$$

The parameter *α* is needed for unknown nodes to provide enough variability in choosing new samples, i.e., the parameter *α* is used to limit a sample *impoverishment* phenomenon. Every time step, the beacon randomly moves a distance *v_b_* in any direction from the previous location. A new sample is generated from each current sample by randomly choosing a point within a circle centered at the current location of the sample and the radius *α* when the *filter(R)* equal to *TRUE*, where *filter(R)* represents the filter condition of real location *R* of the unknown node. The details of condition *filter(R)* will be described in next stage (update stage). In general, the smaller the parameter a, the higher the accuracy of localization is obtained, but the longer time the stable phase of localization process is achieved. The appropriate value *α* is a tradeoff between the high precision of localization and the short period of localization time. Here, the appropriate value *α* could be determined empirically, such as *α=*0.1*r*.

**Update**: In this stage, the unknown node filters the impossible samples based on new observations. The unknown node updates samples as follows:

(23) $$U_{t}=\{l_{t}^{i}|l_{t}^{i}\;\text{where}\;l_{t}^{i}\in P_{t}\;\text{and}\;w(l_{t}^{i})=1\}$$

where *U_t_* represents approximation of posterior density at time *t* after the update stage, and the weight $w(l_{t}^{i})$ will be obtained by $p(o_{t}|l_{t}^{i})$.

In order to state the description of observation $p(o_{t}^{}|l_{t}^{i})$, we define four states for every unknown node during the localization process.

*Outsider*: The unknown node is out of the radio range of the beacon, i.e.,

(24) $$d({\mathrm{beacon}}_{c},R)>r.$$

*Insider*: The unknown node is within the radio range of the beacon, i.e.,

(25) $$d({\mathrm{beacon}}_{c},R)\leq r.$$

*Arriver*: The unknown node receives the current location announcement of the beacon, but did not receive the location announcement from the beacon’s previous location, i.e.,

(26) $$d({\mathrm{beacon}}_{c},R)\leq r\wedge d({\mathrm{beacon}}_{p},R)>r.$$

*Leaver*: The unknown node received the preceding location announcement from the beacon, but does not receive the location announcement from the beacon’s current location, i.e.,

(27) $$d({\mathrm{beacon}}_{c},R)>r\wedge d({\mathrm{beacon}}_{p},R)\leq r.$$

In this paper, we only rely on observations from the beacon. This has two advantages. First, the number of unknown nodes will not affect the accuracy of localization. Second, the computation and communication costs drop drastically, since nodes are no longer involved in the localization of other nodes [9]. There are two possible ways to gather observations from the beacon:

1. Once the unknown node is in Insider state, it gathers this observation, i.e. the filter condition of the real location *R* for any unknown node is:
  (28) $$\mathrm{filter}(R)=\{\begin{array}{c} \mathrm{TRUE}\;\;\;\;\;\;\;\;\;\;\mathrm{if}\;\;\;\;d({\mathrm{beacon}}_{c},R)\leq r\\ \;\mathrm{FALSE}\;\;\;\;\;\;\;\;\;\;\;\;\;\;\;\;\;\;\;\;\;\;\;\;\;\;\;\;\;\;\;\;\;\;\;\;\;\;\;\;\;\;\;\;\;\;\;\;\;\;\;\;\;\mathrm{otherwise}. \end{array}$$
2. When the unknown node is in Arriver or Leaver state, it gathers this observation, i.e. the filter condition of the real location *R* for any unknown node is:
  (29) $$\mathrm{filter}(R)=\{\begin{array}{c} \mathrm{TRUE}\;\;\;\;\;\;\;\;\;\;\mathrm{if}\;\;\;\mathrm{AL}=\mathrm{TRUE}\\ \;\;\mathrm{FALSE}\;\;\;\;\;\;\;\;\;\;\;\;\;\;\;\;\;\;\;\;\;\;\;\;\;\;\;\mathrm{otherwise}, \end{array}$$
  where AL represents Arriver or Leaver state:
  (30) $$\begin{array}{cc} AL & =(d({\mathrm{beacon}}_{c},R)\leq r\wedge d({\mathrm{beacon}}_{p},R)>r)\\ & \vee (d({\mathrm{beacon}}_{c},R)>r\wedge d({\mathrm{beacon}}_{p},R)\leq r). \end{array}$$

From an implementation perspective, in the first way, the beacon just transmits information about its own current location. Once the unknown node hears a location announcement from that beacon, an observation (also called *constraint*) is built to update.

In the second way, the beacon transmits both its current location and its location at the previous time step in each announcement. The unknown node needs to save state (Insider or Outsider) in previous time step with a tag:

(31) $$\mathrm{tag}=\{\begin{array}{c} \mathrm{TRUE}\;\;\;\;\;\;\;\;\;\;\mathrm{if}\;\;\;\mathrm{state}=\mathrm{Insider}\\ \mathrm{FALSE}\;\;\;\;\;\;\;\mathrm{if}\;\;\mathrm{state}=\mathrm{Outsider}. \end{array}$$

This procedure at an unknown node is then as described by Algorithm 1. As can be seen, the tag which represented by variable *StateTag* is initialized to FALSE when the localization started (step 1–3). Then, we get the filter condition of location *R* for an unknown node (step 4–10). Finally, we get *StateTag* with step12–15.

**Algorithm 1.** State at an unknown node.

1:	**If** t=0 **then**
2:	StateTag=FALSE
3:	**end if**
4:	filter(R)=FALSE
5:	**if** Insider ∧ (StateTag=FALSE) **then**
6:	filter(R)=TRUE
7:	**end if**
8:	**if** Outsider ∧ (StateTag=TRUE) **then**
9:	filter(R)=TRUE
10:	**end if**
11:	**if** Insider **then**
12:	StateTag=TRUE
13:	**else**
14:	StateTag=FALSE
15:	**end if**

The first way was adopted by Monte Carlo Localization (MCL) [19] and MSL [9]. As the approach ADO, we adopt second way in the update stage, however. We will evaluate the accuracy of them in Section 4.

Then, the weight of sample is determined by the filter condition:

(32) $$w_{t}=p(o_{t}|l_{t})=\{\begin{array}{c} 1\;\;\;\;\;\;\;\;\mathrm{if}\;\;\;\;\;\mathrm{filter}(R)=\mathrm{TRUE}\\ \;\;\;0\;\;\;\;\;\mathrm{if}\;\;\;\;\;\mathrm{filter}(R)=\mathrm{FALSE}. \end{array}$$

**Resampling**: A common problem with particle filter is the *degeneracy* phenomenon. The degeneracy implies that a large computational effort is devoted to updating particles whose contribution to the approximation to *p*(*l_t_*|*o*_l:*t*_) is almost zero. A suitable measure of degeneracy of the algorithm is the effective sample size *N_eff_* defined as:

(33) $$N_{\mathrm{eff}}=1/\sum_{i=1}^{N}\;{\left(w_{t}^{i}\right)}^{2},$$

notice that *N_eff_* ≤ *N*, and small *N_eff_* indicates severe degeneracy. The method by which the effects of degeneracy can be reduced is to use *resampling* whenever a significant degeneracy is observed (i.e., when *N_eff_* falls below some threshold *N_T_*). The basic idea of resampling is to eliminate particles that have small weights and to concentrate on particle with large weights. We adopt a Systematic resampling algorithm [20] in this paper since it is simple to implement, takes *O(N_s_)* time, and minimizes the Monte Carlo variation.

**Figure 1.**
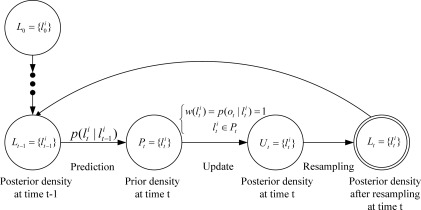
State machine diagram of MBL

Finally, in order to give more clear description of MBL, we describe the main stages as a state machine diagram with labeled transitions, see Figure 1.

### 3.2. A-MBL

#### 3.2.1. Number of Samples

**Figure 2.**
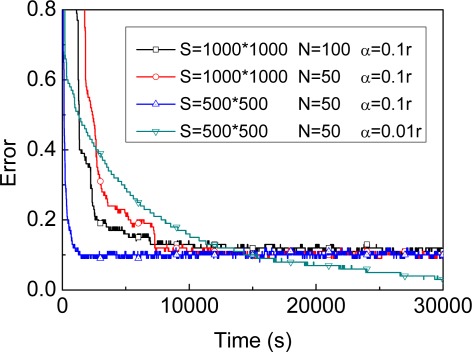
Different exemplars of MBL.

For reference, we first evaluate the trend of location error defined by Equation (35) of four different MBL exemplars (evaluation results shown in Figure 2). Figure 2 shows that, under the same conditions (*S*=1,000×1,000, *α=*0.1*r*), keeping more samples improves efficiency in the beginning of localization. The time complexity of the update stage and the memory requirement to keep samples both are linear in the number of samples needed for the estimation, however. Therefore, the attempt will be made to make more effective use of the available samples, thereby allowing sample sets of reasonable size. As a result, with a fixed number of samples one has to choose a tradeoff so as to allow address efficiency, time and space complexity problem. Before we introduce our method for adaptive particle filter, let us first discuss two existing technique to changing the number of samples during the filter process.

**Figure 3.**
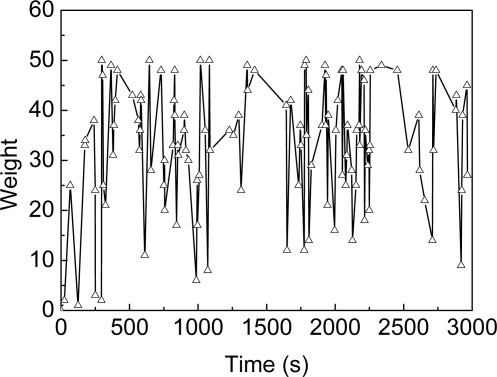
Sum of weight for MBL.

**Likelihood-based adaptation** [21]: The intuition behind this approach is as follows. If the sample set is well in tune with the sensor reading, each individual importance weight is large and the sample set remains small. If we adopt this approach, we will choose smaller sample set than previous when $\sum_{i=1}^{N}\;w_{t}^{i}\geq \mathrm{threshold}$, i.e. the sum of weight $\sum_{i=1}^{N}\;w_{t}^{i}$ has always been greater than a certain value (threshold) since the beginning of a certain time during the localization, the number of sample will be reduced. As shown in Figure 3, the sum of weights for a single unknown node changes over time in MBL. It is clear that more severe fluctuations of weight changing over time in MBL, however. Thus, the conditions of likelihood-based adaptation for MBL shall be deemed invalid.

**KLD-Sampling adaptation** [17]: The key idea of the KLD-sampling approach is to bound the error introduced by the sample-based belief representation. At each iteration, this approach generates samples until their number is large enough to guarantee that the KL-distance between the maximum likelihood estimate and the underlying posterior does not exceed a pre-specified bound. However, the additional cost of KLD-sampling is higher, especially for sensor node, since each lookup takes time logarithmic in the size of the state space. Thus, it is inefficient for us to adopt this approach.

#### 3.2.2. Parameter α

As shown in Figure 2, under the same conditions (*S*=500×500, N=50), a greater value of *α* improves the efficiency in the beginning of localization and improves accuracy at the other extreme. The question is how to determine the value of parameter *α* to achieve both high precision of localization and a short localization time period. We find that the localization error ultimately will remain stable regardless of the value of *α*. Thus, the key idea is to reduce the value of *α* when the stable phase of localization process is achieved, i.e., the dynamic model maintains greater value of *α* in the beginning of localization to achieve shorter localization time and updates to smaller value of *α* to obtain higher precision.

To judge the localization to reach the stable phase, a simple and intuitive approach is to adopt *coefficient of variation c_v_* of recent location estimating results, which is defined as:

(34) $$c_{v}=\frac{\sigma }{\mu },$$

where *σ* and *μ* is the *standard deviation* and the *mean* of recent location estimating results, respectively. When *c_v_*<*ε*, we update *α* by *α=ηα*, where *ε* is a pre-specified threshold and *η* is adjustment factor for *α*. However, when *N* is relatively small, the coefficient of variation is not good to judge the stable phase for a single unknown node, and maintaining recent location estimating results requires additional memory for sensor node. Thus, it is also inefficient for us to adopt this approach.

#### 3.2.3. Our proposed approach and implementation

We adopt two predefined adjustment tables in our approach, one for the number of samples *N*, the other for the parameter α (examples shown in Table 1 and 2). The two tables which include the following fields: TIME, N and TIME, ALPHA. Once some record in the table is matched, the number of samples and the value of *α* in the unknown node will be adjusted according to the corresponding time. The table of *N* and *α* used in this paper was determined empirically. In general, the size of these two tables is very small. Both the implementation and computation overhead of this approach are also small. Thus, we adopt this approach to adapting previous proposed algorithm MBL.

The implementation details are described in Algorithm 2, where *L_N_* is the adjustment list of the number of sample and *L_N_.length* is the length of the list, so does the *L_α_* to the parameter *α*. The beacon transmits the information about these tables when contacting the unknown node for the first time (step 2–6), and the unknown node keeps the value in the list *L_N_* and *L_α_*. Then, the unknown node adjusts the number of samples *N* (step 7–13). In step 8, the *N* which is on the left of assignment denotes new number of samples. The $x_{t}^{i}$ denotes the samples with new *N* in step 11 and the $w_{t}^{i}$ denotes the weights with new *N* in step 12. The parameter *α* (step 14–17) based on above information from the beacon. Finally, the complete A-MBL for every unknown node is shown in Algorithm 3.

**Algorithm 2.** adaptive step in unknown node.

1:	**procedure** ADAPTING
2:	**if** (*d*(*beacon,l*)≤*r*)∧(*firstContracted=FALSE*) **then**
3:	*L_N_* [*k_N_*]← *InitValueFromBeacon*
4:	*L_α_*[*k_α_*]←*InitValueFromBeacon*
5:	*firstContract←TRUE*
6:	**end if**
7:	**if** (*t* = *L_N_*[*k_N_*].*t*) ∧ (*k_N_* < *L_N_*.*length*) **then**
8:	*N_p_ ← N*
9:	*N ← L_N_* [*k_N_*].*N*
10:	*k_N_ ← k_N_* +1
11:	$x_{t}^{i}\leftarrow x_{t}^{j}(i\leftarrow 1,N;j\leftarrow 1,N_{p})$
12:	$w_{t}^{i}\leftarrow w_{t}^{j}(i\leftarrow 1,N;j\leftarrow 1,N_{p})$
13:	**end if**
14:	**if** (*t* = *L_α_*[*k_α_*].*t*) ∧ (*k_α_* < *L_α_*.*length*) **then**
15:	*α* ← *L_α_*[*k_α_*].*α*
16:	*k_α_* ← *k_α_* + 1
17:	**end if**
18:	**end procedure**

**Algorithm 3.** A-MBL.

1:	*k_α_* ← 0
2:	*k_N_* ← 0
3:	*firstContracted* ← *FALSE*
4:	**for***i* ← 1, *N***do**
5:	*INITIALIZATION*
6:	**end for**
7:	**for***t* ← 1,*T***do**
8:	**for***i* ← 1, *N***do**
9:	$l_{t}^{i}∼p(l_{t}^{i}|l_{t-1}^{i})$
10:	$w_{t}^{i}\leftarrow p(o_{t}|l_{t}^{i})$
11:	**end for**
12:	$w_{t}^{i}\leftarrow \mathrm{NORMALIZE}(w_{t}^{i})$
13:	$N_{\mathrm{eff}}\leftarrow \mathrm{NEFF}(w_{t}^{i})$
14:	**if** (*N_eff_* < *N_T_* ∧ *filter*(*l*) = *TRUE*) **then**
15:	$\left\{<l_{t}^{j},w_{t}^{j}>\}\leftarrow \mathrm{RESAMPLING}(\{<l_{t}^{i},w_{t}^{i}>\}\right)$
16:	**end if**
17:	*ADAPTING*
18:	**end for**

## 4. Evaluation

### 4.1. Assumption

The key metric [9] for evaluating a localization algorithm is the accuracy of the location estimates or *localization error*. This is computed as follows:

(35) $$\mathrm{Error}=\frac{1}{M}\sum_{i=1}^{M}\;∥e_{i}-R_{i}∥,$$

where *M* is the number of unknown nodes, *R_i_* denotes the real location of the *i*-th unknown node, *e_i_* denotes the location estimate of the *i*-th unknown sensor where *i=*1*,…,M* and □*e_i_* − *R_i_*□ denotes the distance between locations *e_i_* and *R_i_*. The errors shown in the simulation results are in terms of the radio range, i.e., the errors shown are computed by dividing the error in Equation (35) by the radio range of sensors. Most parameter settings for our simulations are those used in [19, 9]. Our results were obtained using sensors randomly distributed in a 500 units × 500 units square field, i.e. S=500×500. In our experiments, we set ideal radio range *r=*100, the number of unknown nodes *M=*100, the number of samples for an unknown node *N=*50, the parameter α=0.1r in the prediction stage, the maximum speed of beacon *v_max_=*1.0*r* and two predefined adjustment tables (shown in Table 1 and 2) for adapting unless otherwise specified. Other simulation parameters of the unknown node are based on the MicaZ sensor node. The beacon’s movement is implemented using random waypoint mobility model.

In this section, we first evaluate MBL algorithm under various parameters configuration, such as the maximum speed of the beacon, the number of samples for an unknown node, and the impact of parameters *α* in the prediction stage. Then, under the same conditions (e.g. only use a single mobile beacon for localization), we compare the efficiency and accuracy of MBL, A-MBL, MSL, and ADO in different parameters configuration. In addition, we will consider a noisy environment with random noise added to measurements.

### 4.2. Parameters of MBL

**Figure 4.**
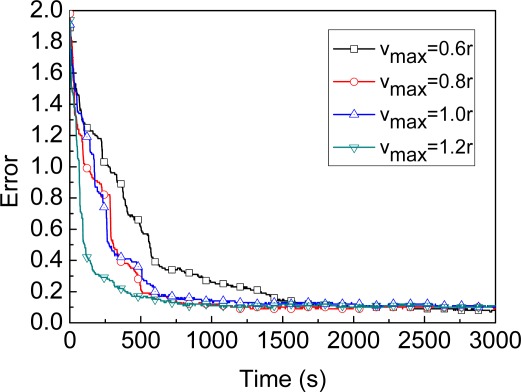
Location convergence.

**Maximum speed of the beacon**: Figure 4 shows the convergence of MBL algorithm under four different *v_max_* scenarios. In general, the faster the speed of the beacon, the quicker the stable phase is reached. Because the faster the speed of the beacon, the more the number of unknown nodes which the beacon could contact with. When *v_max_* is greater than or equal to 0.6*r*, the convergence of MBL is particularly fast, and the localization process can be divided into the initialization phase and the stable phase. In the initialization phase, the estimate error decreases dramatically as new observations (the localization is improved by both the current observation and previous observations) are incorporated. In the stable phase, the impact of filter and the beacon’s mobility reach some balance, and the estimate error fluctuates around a minimum value. When *v_max_* is greater than or equal to 0.8*r*, the curves of convergence (e.g. *v_max_=*1.0*r* and *v_max_=*1.2*r* shown in Figure 4) are very close to each other. Based on above comparison, we set *v_max_=*1.0*r* as the default parameters configuration. When the time is greater than 1000 under this default parameters configuration, the error fluctuates slightly about a constant value (nearly 0.1).

**Figure 5.**
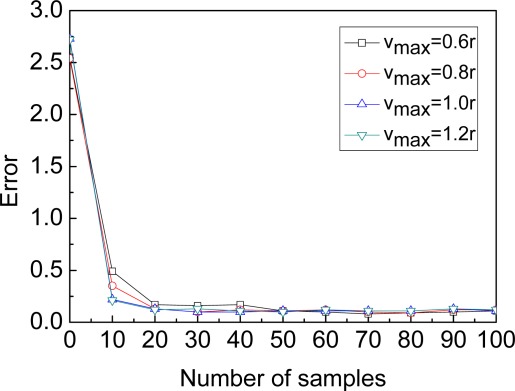
Impact of sample size.

**Number of samples for MBL**: Maintaining more samples for the MCL algorithm can improve accuracy, but requires additional memory [19]. Based on this comment, we should select an appropriate number of samples which does not affect the accuracy of localization and also does not waste memory either. Figure 5 shows the impact of sample size on location accuracy. The estimate error drops rapidly at the beginning, since a small number of samples cannot adequately reflect the probability distribution. The estimate error is fairly stable after sample size 50 and the accuracy improves only minimally by increasing the number of samples to 100. Hence, MBL is efficient in both memory and computation when the number of sample is close to 50. So, we choose 50 as default sample quantity to save memory and achieve good accuracy.

**Figure 6.**
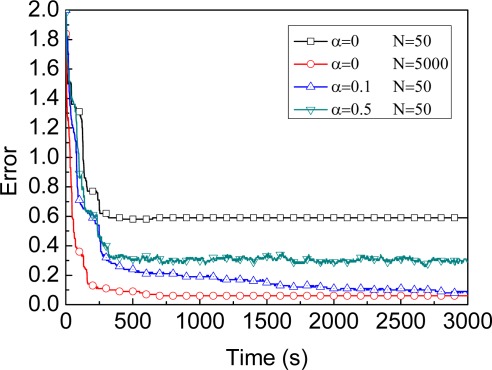
Impact of parameter α.

**Parameter** *α* **for MBL**: Figure 6 shows the impact of parameter *α* on location accuracy. If *α* = 0, i.e., all samples of the unknown node always keep static in the prediction stage. As a result, it cannot provide enough variability in choosing new samples. Hence, to improve the accuracy, the algorithms should increase the number of samples for each unknown node. In Figure 6, when *α=*0, the number of samples is needed to reach about 5,000 in order to achieve the similar precision as *α=*0.1*r* (but this number of samples is just 50). The parameter *α* significantly improves the accuracy and reduces the number of samples. Based on a number of experimental results, we adopt *α=*0.1*r* and the number of samples *N*=50 for an unknown node as default values.

### 4.3. Comparison of Different Algorithms

In order to compare different algorithms under the same conditions, MSL in our evaluation will only use a single mobile beacon for localization.

**Figure 7.**
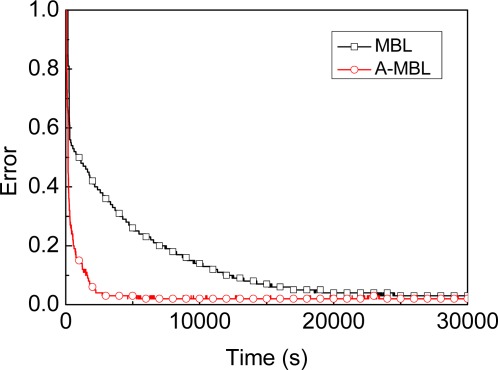
Comparison of efficiency.

**Efficiency**: The graph in Figure 7 shows the efficiency comparison for MBL (*α*=0.01*r*), A-MBL under same conditions except the parameter *α*. In order to obtain close precision, MBL spends more time with a fixed *α* than A-MBL with adapting *α* obtained from predefined adjustment tables.

**Figure 8.**
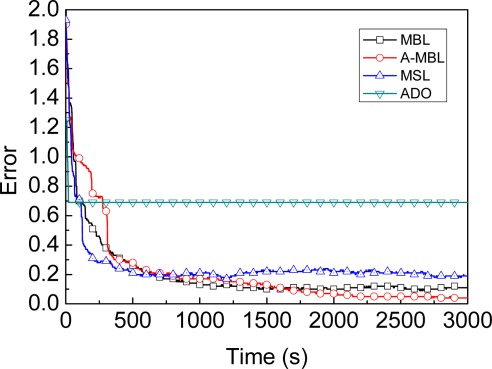
Comparison of accuracy.

**Accuracy**: Figure 8 shows the comparison of accuracy for MBL, A-MBL, MSL and ADO under same maximum speed of the beacon (*v_max_*). Figure 8 illustrates that the curve of ADO drops to a certain value, then to the horizon, because the Arriver and Leaver information only be used once by ADO when the beacon passes by traverse route. Even though the beacon in ADO pass by the radio ranges of the unknown node on many occasions, the accuracy of unknown node will not be improved. Thus, as the time goes on, the average accuracy of all unknown nodes does not decrease. As shown in Figure 8, MBL and MSL, these two curvature of the curves are very similar to each other. As the time goes on, these two curves both reach to the stable phase. MBL shows nearly 50% better performance and nearly equal time of localization when compared to the MSL. Before the parameter *α* in A-MBL adjusted, the accuracy of MBL and A-MBL are very close. Once the parameter *α* in A-MBL has been adjusted, the accuracy of A-MBL is further improved. The accuracy of MBL still keeps stable, however. Thus, it can be seen from the Figure 8 that A-MBL outperforms MBL, MSL and ADO.

**Figure 9.**
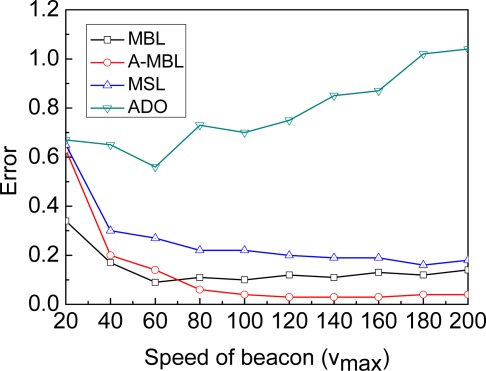
Speed of beacon.

**Speed of the beacon**: Figure 9 shows how the localization error varies with the changing speed of the beacon. As a result, the faster speed of the beacon, the worse accuracy of ADO is achieved. Because the faster speed of the beacon which just travels the whole deployment area of a sensor network only once, the more the numbers of unknown nodes do not be localized. With further increase in the speed of beacon, the accuracy of MBL is close to MSL, for the area of Arriver and Leaver will become smaller. Finally, when *v_b_* ≥ 2*r*, the area of Arriver and Leaver is equal to zero, and then MBL will degrade to MSL. The emergence of the significant error when the beacon (*v_max_*=20) at low speed, just because the time (*t*=3,000) is too short for convergence of MBL, A-MBL, and MSL. As a result of Figure 9, A-MBL always performs better than both MBL and MSL in some range of speed.

**Figure 10.**
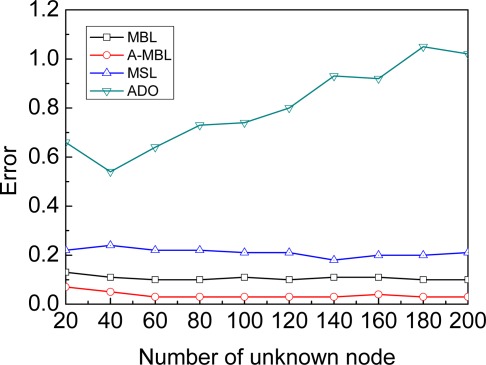
Number of unknown nodes.

**Number of the unknown nodes**: In this experiment we vary the number of unknown nodes from 20 to 200. We set the time of beacon movement as 3,000 to achieve the stable phase. As shown in Figure 10, the number of unknown nodes will not affect the accuracy of A-MBL, MBL and MSL, just because each of them only use the beacon for localization in our evaluations (not from neighborhood information).

### 4.4. Irregularity

All of our previous experiments assumed an ideal scenario where location sensory data are not influenced by irregular radio range and any receiver within the radio range of sender will hear the packets from that sender. However, on the one hand, variability in actual radio transmission patterns can have a substantial impact on localization accuracy depending on the localization technique [19]. On the other hand, the packet reception depends not only on the sender, but also on the receiver.

**Figure 11.**
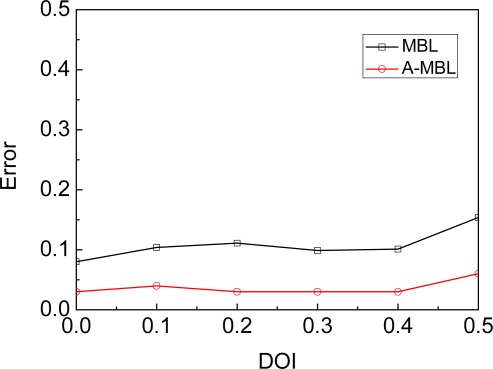
Impact of Irregularity.

Figure 11 shows the impact of degree of irregularity on estimate error. The MBL and A-MBL are not substantially affected. We use degree of irregularity (DOI) to denote the maximum radio range variation in the direction of radio propagation. For example, if DOI = 0.1, then the actual radio range in each direction is randomly chosen from [0.9r, 1.1r].

If the unknown node (receiver) within radio range of the beacon (sender) will not hear a location announcement (such as network collisions or missed packages) from that beacon at some time, the beacon must keep moving for longer time to send location information which the unknown node could receive them, i.e. the time to achieve final stable phase of accuracy as ideal state will be extended. The accuracy of MBL and A-MBL will not be affected in such scenarios.

## 5. Related Works

In this section, we provide a brief survey focusing on mobile-assisted localization approaches suitable for WSNs. MAL [11] proposed by Priyantha *et al.* involves a mobile-assisted localization method which employs a mobile user to assist in measuring distances between node pairs until these distance constraints form a “globally rigid” structure that guarantees a unique localization. In [10], Kim *et al.* propose a novel range-based localization scheme which involves a movement strategy with a low computational complexity of mobile beacon, called mobile beacon-assisted localization (MBAL).

Different from above range-based deterministic mobile beacon-assisted approaches, many range-base probabilistic approaches have been proposed. Sichitiu *et al.* [12] propose a radio frequency (RF) based (i.e. the received signal strength indicator (RSSI) is used for ranging) localization method using Bayesian inference for processing information from one mobile beacon. Caballero *et al.* [22] present range-based (which process the RSSI value in each node in order to localize the nodes of a static wireless network) methods for the 3D localization of an outdoor WSN by using a single flying beacon-node on-board an autonomous helicopter. The technique is based on particle filtering and allows a distributed computation of the position of the nodes. Marinakis *et al.* [23] present, based on Markov Chain Monte Carlo (MCMC) methodology, a hybrid Inference for Sensor Network Localization using a Mobile Robot, However, this method requires a special hardware platform for the above inference technique: a mobile robot is observed by one of the component stationary cameras in a sensor network. Ihler *et al.* [24] present and demonstrate range-based (scenarios in which each sensor is equipped with a wireless and/or acoustic transceiver and distance is estimated by received signal strength or time delay of arrival between sensor locations) the utility of nonparametric belief propagation (NBP), a generalization of particle filtering, for both estimating sensor locations and representing location uncertainties. Peng *et al.* [14] propose a range-based (which measure the RSS at different distances between a transmitter and a receiver pair) probabilistic, constraint-based approach robust to range measurement inaccuracies.

All above range-based approaches are constrained by the expensive cost and high energy consumptions of the ranging hardware devices. Furthermore, in many practical situations, the measurements are far from accurate (and even sometimes unobtainable) due to highly dynamic environments [25].

Due to the hardware limitations and energy constraints of sensor nodes, range-free localization approaches are cost-effective alternatives to range-based approaches. Walking GPS [26] is a range-free localization, in which the deployer (either person or vehicle) carries a GPS device that periodically broadcasts its location. Each node computes its location estimate according to either the broadcasting positions of the moving beacon or the positions of its neighbors. ADO provides a distributed method to localization of sensor nodes using a single moving beacon where sensor nodes compute their position estimate based on the range-free technique. The method uses the arrival and departure information of a walking beacon.

Our proposed method differs significantly from previous range-base or range-free mobile-assisted localization works because we adopt range-free techniques and solve the problem from particle filter perspective.

Our work is similar to that of Hang *et al.* [13], which discusses the Monte Carlo sampling steps in the context of the localization using a single beacon for various types of observations such as ranging, Angle of Arrival (AoA), connectivity and combinations of those. This method works more like an online algorithm, in which all computation is done at the beacon.

Coates *et al.* [27] present two distributed particle filtering algorithms for Sensor Networks. Different from our work which locate static unknown nodes, this approach is used to track posterior distributions in Markovian state-space models using sensor networks.

The range-free algorithm MCL proposed by Hu *et al.* only works in mobile sensor networks. MCL works well in mobile sensor networks as long as the speed of movement is not very low. The paper proposes two possible filter approaches. The first approach adopts Arriver and Leaver information (i.e. beacon transmits both its current location and its location at the previous time step in each announcement). The second adopts the current neighborhood (beacons and unknowns nodes) information. The other range-free algorithm called MSL proposed by Rudafshani *et al*., works well when some or all nodes are static or mobile. Unlike MCL in the prediction stage, the parameter *α* is needed for MSL to work well when no sensors move. Each node maintains a set of weighted samples denoting its possible locations and its weight is determined using the current neighborhood information. Though these approaches are not especially for a single mobile-assisted sensor networks, our work is inspired by above approaches. In the prediction stage, we adopt the parameter *α* proposed by MSL. In the update stage, we adopt Arriver and Leaver information proposed by MCL.

## 6. Conclusions

In this paper, we propose two range-free, distributed and probabilistic mobile beacon-assisted localization approaches for static WSNs, MBL and A-MBL. Evaluation results show that the accuracy of A-MBL outperforms MBL, MSL and ADO in static WSNs when all of them use only a single mobile beacon for localization. As future work, two new issues will be considered. First, whether the use of information from unknown nodes (especially the neighbor nodes) which may have greater communication cost to increase efficiency and accuracy in mobile beacon-assisted localization needs further research. Second, though adopting predefined adjustment tables in A-MBL is convenient and effective, obtaining these tables is difficult. We will also consider some of self-adaptive mechanism in our approaches to achieve more flexibility.

## Figures and Tables

**Table 1:** Predefined adjustment tables for N.

**ID**	**Time**	**N**
1	0	50
2	2,000	20

**Table 2:** Predefined adjustment tables for α.

**ID**	**Time**	**Alpha**
1	0	0.1
2	1,500	0.01
